# Three-Dimensional Cell Metabolomics Deciphers the Anti-Angiogenic Properties of the Radioprotectant Amifostine

**DOI:** 10.3390/cancers13122877

**Published:** 2021-06-09

**Authors:** Theodora Katsila, Styliani A. Chasapi, Jose Carlos Gomez Tamayo, Constantina Chalikiopoulou, Eleni Siapi, Giorgos Moros, Panagiotis Zoumpoulakis, Georgios A. Spyroulias, Dimitrios Kardamakis

**Affiliations:** 1 Institute of Chemical Biology, National Hellenic Research Foundation, 11635 Athens, Greece; cchalikio@eie.gr (C.C.); esiapi@eie.gr (E.S.); geomoro@hotmail.gr (G.M.); pzoump@eie.gr (P.Z.); 2 Department of Radiation Oncology, University of Patras Medical School, 26504 Patras, Greece; kardim@upatras.gr; 3 Department of Pharmacy, University of Patras, 26504 Patras, Greece; stella.chimic@gmail.com (S.A.C.); G.A.Spyroulias@upatras.gr (G.A.S.); 4 GRIB (IMIM/UPF), PRBB, E-08003 Barcelona, Spain; josecarlos.gomez@upf.edu

**Keywords:** amifostine, 3D cellular angiogenesis assay, metabolomics, drug repurposing, radioprotection, anti-angiogenesis

## Abstract

**Simple Summary:**

Cancer and inflammation share aberrant angiogenesis as a hallmark, and, thus, anti-angiogenetic strategies remain of key interest. Amifostine, which is already a drug on the market, may be of further benefit to patients also in the context of drug repurposing. To shed light on the anti-angiogenic properties of amifostine during human adult angiogenesis and grasp the early events of angiogenesis, we employed 3D cell untargeted metabolomics by liquid chromatography–mass spectrometry and nuclear magnetic resonance spectroscopy in the presence of vascular endothelial growth factor-A or deferoxamine (pro-angiogenic factors that exhibit distinct angiogenesis induction profiles). Our findings reveal mechanism-specific inhibitory profiles of amifostine against VEGF-A- and deferoxamine-induced angiogenesis. Amifostine may serve as a dual radioprotective and anti-angiogenic agent in radiotherapy patients.

**Abstract:**

Aberrant angiogenesis is a hallmark for cancer and inflammation, a key notion in drug repurposing efforts. To delineate the anti-angiogenic properties of amifostine in a human adult angiogenesis model via 3D cell metabolomics and upon a stimulant-specific manner, a 3D cellular angiogenesis assay that recapitulates cell physiology and drug action was coupled to untargeted metabolomics by liquid chromatography–mass spectrometry and nuclear magnetic resonance spectroscopy. The early events of angiogenesis upon its most prominent stimulants (vascular endothelial growth factor-A or deferoxamine) were addressed by cell sprouting measurements. Data analyses consisted of a series of supervised and unsupervised methods as well as univariate and multivariate approaches to shed light on mechanism-specific inhibitory profiles. The 3D untargeted cell metabolomes were found to grasp the early events of angiogenesis. Evident of an initial and sharp response, the metabolites identified primarily span amino acids, sphingolipids, and nucleotides. Profiles were pathway or stimulant specific. The amifostine inhibition profile was rather similar to that of sunitinib, yet distinct, considering that the latter is a kinase inhibitor. Amifostine inhibited both. The 3D cell metabolomics shed light on the anti-angiogenic effects of amifostine against VEGF-A- and deferoxamine-induced angiogenesis. Amifostine may serve as a dual radioprotective and anti-angiogenic agent in radiotherapy patients.

## 1. Introduction

Today, technological advances and voluminous datasets are used to inform biomedical discoveries and validate hypotheses. State-of-the-art approaches coupled with information technologies are also of prime interest toward drug repositioning and pharmaceutical marketing [1,2]. We aim to translate information growth into knowledge growth.

Amifostine (WR-2721, Ethyol^®^) clinically acts as a radioprotector and cytoprotector for normal tissues in patients under several anti-cancer therapies [3]. It is a phosphorylated aminothiol prodrug that is converted to its active thiol metabolite WR-1065 upon the action of alkaline phosphatase, an enzyme found on the cell membranes of epithelial or endothelial tissues [4]. Amifostine selectively protects normal tissues due to (i) the higher concentration of alkaline phosphatase on normal cells, (ii) the diminished vascular supply of the drug to tumor tissues, and (iii) the neutral environment (preferably pH 7.4) on normal tissues in comparison with the acidic environment on tumors that reduces the activation of the prodrug (dephosphorylation) [5].

Several studies have reported various cytoprotection mechanisms of amifostine, such as free radical scavenging [5], reduced oxygen consumption in normal cells [6,7], DNA protection from fragmentation or other effects [8], boosting DNA repair [9], and anti-mutagenic effects [10]. It has also been found that amifostine protects blood vessels from the effects of ionizing radiation [11,12] and inhibits angiogenesis in vitro and in vivo (chick embryo chorioallantoic membrane angiogenesis model, CAM model) [13,14,15].

Angiogenesis refers to the complex process of generating new blood cells from the existing vasculature [16]. Angiogenesis growth factors and cytokines are tightly connected with the regulation of angiogenesis. Vascular endothelial growth factor-A (VEGF-A), as one of the most studied growth factors, induces tube formation, migration, and cell sprouting [17]. Several in vitro and in vivo assays have been developed to allow for a holistic approach to study angiogenesis, but no single angiogenic assay can elucidate the entire process, as many molecular mechanisms involved in angiogenesis are still poorly understood [18,19]. Even the CAM model, although informative, is inadequate to fully recapitulate human adult angiogenesis [20].

**Figure 1:**
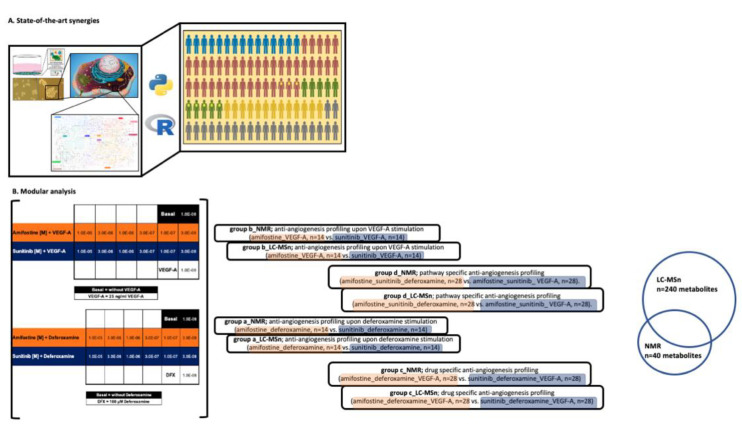
A schematic representation of our strategy. (**A**) State-of-the-art synergies. A 3D cellular angiogenesis assay that mimics human adult angiogenesis in vivo as well as the first stages of the angiogenic cascade was coupled to state-of-the-art technologies (LC-MS and NMR spectroscopy) and chemo-informatics tools/databases to assess the anti-angiogenic action of amifostine in a human adult angiogenesis setting. (**B**) Modular analysis (*n* = 3 cell lines, *n* = 2 cell culture conditions, *n* = 32 test conditions, *n* = 3 biological replicates (per test condition), and *n* = 3 technical replicates (per test condition)). Group comparisons are color-coded; orange, angiogenesis stimulation and blue, angiogenesis inhibition. A total of *n* = 240 metabolites were identified and quantified by LC-MS-based analysis, whereas 40 metabolites were identified and quantified from the 1H CPMG NMR spectra. A total of 36 metabolites were identified and quantified by both LC-MS and NMR spectroscopy.

Three-dimensional biology and cell culture represent a more realistic approach and better recapitulate human physiology [21,22]. The 3D cellular angiogenesis assays in vitro have been known to mimic human tissue architecture and adult angiogenesis in vivo, as well as the first stages of the angiogenic cascade [23]. Herein, a 3D cellular angiogenesis assay is coupled to untargeted metabolomics by liquid chromatography–mass spectrometry (LC-MS) and nuclear magnetic resonance (NMR) spectroscopy to explore further the anti-angiogenic action of amifostine (Figure 1A,B) [24,25].

## 2. Materials and Methods

Cell Culture: HUVEC, human umbilical vein endothelial cells (PromoCell, Heidelberg, Germany; passage 3 to 4; pooled donors), CRL2922, and CRL2873 cell lines were tested in 2D and 3D cell culture conditions. All cell lines chosen, being capable of 2D and 3D cell culture conditions, are well established in vitro models for (anti)angiogenesis studies. Cells were cultured in 5% CO_2_ and 95% humidified atmosphere air at 37 °C in endothelial cell growth and basal medium MV (ECGM/ECBM, PromoCell). Spheroids (hanging drops) were prepared on plastic dishes to allow overnight spheroid aggregation [26,27]. Next, spheroids were seeded in 0.9 mL of a collagen gel and pipetted into individual wells of a 24-well plate to allow polymerization.

Cell sprouting and scattering for IC_50_ calculations: Cell sprouting is quantified by measuring the sprout length, and next, the cumulative sprout length (CSL) is determined. The sprouting intensity was quantitated by an image analysis system determining CSL per spheroid using an inverted microscope and the digital imaging software NIS-Elements BR 3.0 (Nikon). The mean CSL of randomly selected spheroids (*n* = 10) was analyzed as an individual data point. For IC_50_ calculations, raw data were converted into percent (%), with cell sprouting compared to the solvent control (set to 100%) and the basal control (without stimulation), which was set to 0%. IC_50_ values were determined using GraphPad Prism Mac 5.0f software with constraint of the bottom set to 0 and the top set to 100, using a nonlinear regression curve fit with variable hill slope. The equation is a four-parameter logistic equation.

Treatment regimen: VEGF-A or deferoxamine in 0.1% DMSO (stimulants to angiogenesis) was added to spheroids. After 30 min, amifostine in H_2_O was added. Sunitinib in 0.1% DMSO, an angiogenesis inhibitor, served as a reference compound. All compounds were tested in the same concentration range from 1.0 × 10^−5^ M to 1.0 × 10^−8^ M for all individual data points (namely 1.0 × 10^−5^, 3.0 × 10^−6^, 1.0 × 10^−6^, 3.0 × 10^−7^, 1.0 × 10^−7^, 3.0 × 10^−8^, and 1.0 × 10^−8^ M). Plates were incubated at 37 °C for 24 h and fixed by adding 4% PFA (Roti-Histofix, Roth, Karlsruhe, Germany). Additional plates with identical treatments were frozen without fixation at −80 °C for LC-MS- and NMR-based untargeted metabolomics.

Sample preparation for cell pellet DNA assay: Cells were seeded in 10 cm dishes in triplicate and were incubated for 4 h at 37 °C and 5% CO_2_. Then, they were washed twice with dry ice-cooled 60% methanol containing 0.85% (*w*/*v*) ammonium bicarbonate and extracted with 500 μL of dry ice-cooled methanol/chloroform/water (7:2:1) (*v*/*v*/*v*). The cells were scraped and transferred into a microfuge tube on ice and vortexed for 30 s. Following centrifugation for 2 min at ×20,000 *g* at 4 °C, the pellet was air-dried at room temperature and then used for DNA extraction [28]. DNA extraction was carried out with a genomic DNA mini kit for blood/cultured cells (QIAGEN), according to the manufacturer’s instructions. DNA concentrations were measured using a UV–Vis spectrophotometer (Quawell Q5000). The lysate of this step was used for untargeted metabolomic data analysis.

Sample preparation for metabolomic analysis: For LC-MS- and NMR-based untargeted metabolomics, the additional plates with identical treatments, which were frozen without fixation at −80 °C till further analysis, were thawed overnight at 4 °C. Next, samples were filtered using an Amicon Ultracel-3K filter (Millipore Corporation). For LC-MS-based untargeted metabolomic analysis, sample extraction was carried out by adding ice-cold methanol (3:1) (*v*/*v*), and then the mixture was shaken at 4 °C for 15 min. Samples were then centrifuged at ×14,000 *g* for 10 min and supernatants were collected ready for analysis. For NMR-based untargeted metabolomic analysis, the frozen lyophilized cell extracts were thawed at room temperature. Each extract was mixed with 540 μL of D_2_O resulting in a sample total volume of 600 μL, after the addition of 60 μL DSS (0.1 mM) solution used as an internal standard chemical shift reference. Samples were vortexed briefly and transferred to a 5 mm NMR tube (Bruker BioSpin) ready for analysis.

LC-MS-based untargeted metabolomic analysis: The analysis was performed on an LTQ-Orbitrap Velos mass spectrometer (Thermo Fisher Scientific, Bremen, Germany) connected to an Accela ultra-high-performance LC (UHPLC) system. A Kromasil Etermity C18 column (100 Å, 2.5 um, 100 mm × 2.1 mm) was used. To monitor the instrument performance over time and chromatographic integrity, including retention time shifts, quality control samples were prepared as a mix of each sample. The injection volume was set at 5 μL, and the mobile phase flow rate was set at 0.2 mL/min. Mobile phase solvents were A (95% H_2_O, 5% methanol, 10 mM tributylamine, 9 mM acetate, pH = 8.2) and B (100% methanol). The eluting gradient program was the following: 0–2.5 min (95% A, 5% B), 2.5–5.0 min (80% A, 20% B), 5.0–7.5 min (80% A, 20% B), 7.5–13.0 min (55% A, 45% B), 13.0–15.5 min (5% A, 95% B), 15.5–18.5 min (5% A, 95% B), 18.5–19.0 min (95% A, 5% B), and 19.0–25.0 min (95% A, 5% B). Data were processed with Xcalibur software (version 2.1, Thermo Scientific, Waltham, MA, USA).

NMR-based untargeted metabolomic analysis: The ^1^H-NMR spectra were recorded at 298K on a Bruker Avance III HD 700 MHz NMR spectrometer equipped with a cryogenically cooled 5.0 mm ^1^H/^13^C/^15^N/D Z-gradient probe. For each sample, a mono-dimensional (1D) ^1^H NMR spectrum was acquired using a standard Carr–Purcell–Meiboom–Gill (cpmgpr1d.comp; Bruker BioSpin) pulse sequence to remove any broad signal contribution imposing a T2 filter. All monodimensional spectra were acquired with 256 scans, a relaxation time of 4 s, a receiver gain of 50.8, 256 scans, and a spectral width of 12.9814 ppm. A 2D ^1^H J-resolved (jresgpprqf.comp; Bruker BioSpin) pulse sequence was also performed for metabolite identification separating chemical shifts and J couplings into two different spectral dimensions. Two-dimensional ^1^H-^13^C HSQC NMR spectra were applied for accurate assignment and also when highly overlapping spectral regions were present in the monodimensional spectra. For all samples, matching and shimming were performed manually.

Data processing and statistical analysis: For both LC-MS-based [29] and NMR-based [30,31] approaches, an untargeted analytical approach was employed, and, hence, univariate as well as multivariate statistical methods were applied coupled to both unsupervised and supervised methods. Multiple data comparisons were performed as follows to shed light on amifostine’s 3D cell anti-angiogenesis metabolome: (a) anti-angiogenesis profiling upon deferoxamine stimulation (amifostine_deferoxamine, *n* = 14 vs. sunitinib_deferoxamine, *n* = 14), (b) anti-angiogenesis profiling upon VEGF-A stimulation (amifostine_VEGF-A, *n* = 14 vs. sunitinib_VEGF-A, *n* = 14), (c) drug-specific anti-angiogenesis profiling (amifostine_deferoxamine_VEGF-A, *n* = 28 vs. sunitinib_deferoxamine_VEGF-A, *n* = 28), and (d) pathway-specific anti-angiogenesis profiling (amifostine_sunitinib_deferoxamine, *n* = 28 vs. amifostine_sunitinib_ VEGF-A, *n* = 28).

For LC-MS-based untargeted metabolomics, data processing and analysis was performed using MetaboKit, MetaboAnalyst 4.0 and R 3.2.2 [32,33,34]. Metabolites present in more than 33% of the samples were included in the analyses. After removing uninformative features, the resulting number of metabolites was decreased drastically to ~1/4. For those metabolites passing the criteria, empty values were annotated with a small value (1). Data centering and unit variance scaling were performed. Log2fold calculation, principal component analysis (PCA), partial least squares (PLS), and partial least squares discriminant analysis (PLS-DA) analyses were performed for every comparative analysis. PLS VIP (variable importance in projection) values were determined. Only metabolites with a log2fold ≥ 2 were selected for subsequent enrichment analysis. Enrichment analysis was performed using MetaboAnalyst 4.0 employing pathway-associated metabolite sets (SMPDB) [33,34].

For NMR-based untargeted metabolomics, data processing was performed after free induction decays were multiplied by an exponential function equivalent to a 1.0 Hz line-broadening factor before applying Fourier transform. Transformed NMR spectra were phased, baseline corrected, and calibrated (using as a reference peak the 1H signal of the anomeric hydrogen of a-glucose doublet at 5.24 ppm) manually using Topspin 3.2 (Bruker BioSpin). Each processed CPMG spectrum (0.20–10.00 ppm) was uniformly segmented into 0.02 ppm spectral width buckets in Amix 3.9.13 software (Bruker Biospin). Spectral regions for residual water (6.00–4.50 ppm) or glycerol resonating at 3.65–3.61 (dd) ppm, 3.57–3.52 ppm (dd), and 3.78–3.75 (m) ppm were excluded, reducing the system to 403 bins. Datasets were analyzed by SIMCA^®^16, MetaboAnalyst 4.0 software and R 3.2.2 [33,34]. Metabolite identification was performed using Chenomx NMR software (Profiler 8.1 Chenomx Inc., Canada), the human metabolome database (HMDB), and data from the literature [35,36,37,38].

## 3. Results

### 3.1. Amifostine Inhibits VEGF-A- and Deferoxamine-Induced Cell Sprouting

Angiogenesis in spheroids was stimulated by either VEGF-A or deferoxamine to test (i) if amifostine can exert inhibitory effects and (ii) which molecular pathways are involved. Sunitinib served as a reference standard to determine the range of such inhibition by amifostine. The sprouting intensity was quantitated for cumulative sprout length (CSL) determination. The mean CSL of randomly selected spheroids (hanging drops, *n* = 10) was analyzed as an individual data point.

**Figure 2:**
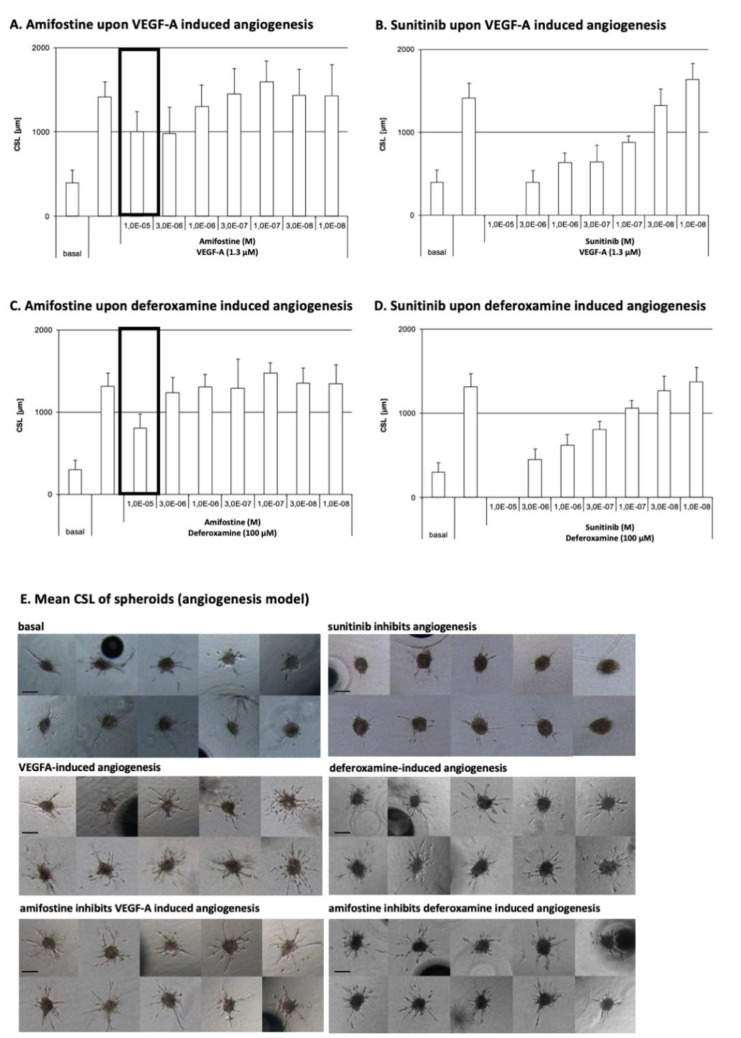
Amifostine effects on VEGF-A- or deferoxamine-induced angiogenesis. (**A**) Amifostine effects on VEGF-A-induced spheroid sprouting; (**B**) sunitinib effects on VEGF-A-induced spheroid sprouting; (**C**) amifostine effects on deferoxamine-induced spheroid sprouting; (**D**) sunitinib effects on deferoxamine-induced spheroid sprouting; (**E**) mean CSL of spheroids (**A**–**D**, mean values and SD of CSL of 10 randomly selected spheroids per data point are depicted; 10× objective magnification, digital imaging software Cell-Olympus, Hamburg, Germany). Drug-dependent alterations in the sprouting intensity were ranked from zero (0, no or strongly reduced sprouting activity compared with baseline) to triple cross (+++, enhanced sprouting activity compared with baseline). Herein, sunitinib corresponds to zero (0) and amifostine to inhibition (+).

Amifostine was found to inhibit VEGF-A- or deferoxamine-induced sprouting angiogenesis and, hence, angiogenesis with an IC_50_ of 1.0 × 10^−^^5^ M. Sunitinib has an IC_50_ of 1.2 × 10^−^^7^ M and 3.4 × 10^−^^7^ M for VEGF-A- or deferoxamine-induced sprouting, respectively. Overall data are depicted in Figure 2.

### 3.2. LC-MS- and NMR-Based 3D Cell Metabolomes Reveal the Anti-Angiogenic Profile of Amifostine

Data obtained from both state-of-the-art analytical approaches (LC-MS, NMR spectroscopy) agreed on the anti-angiogenic action of amifostine upon VEGF-A- or deferoxamine-induced spheroid sprouting, mimicking the inhibitory effects of sunitinib.

A total of n = 32 metabolomes were acquired to allow for the following group comparisons (Figure 1B): (a) anti-angiogenesis profiling upon deferoxamine stimulation (amifostine_deferoxamine, n = 14 vs. sunitinib_deferoxamine, n = 14), (b) anti-angiogenesis profiling upon VEGF-A stimulation (amifostine_VEGF-A, n = 14 vs. sunitinib_VEGF-A, n = 14), (c) drug-specific anti-angiogenesis profiling (amifostine_deferoxamine_VEGF-A, n = 28 vs. sunitinib_deferoxamine_VEGF-A, n = 28), and (d) pathway-specific anti-angiogenesis profiling (amifostine_sunitinib_deferoxamine, n = 28 vs. amifostine_sunitinib_ VEGF-A, n = 28). A total of 240 metabolites were identified and quantified by the LC-MS-based analysis, whereas 40 metabolites were identified and quantified from the ^1^H CPMG NMR spectra. A total of 36 metabolites were identified and quantified by both LC-MS and NMR spectroscopy (Supplementary Tables S1 and S2).

Overall, all comparisons confirmed the anti-angiogenic action of amifostine, also suggesting the top metabolites and molecular pathways per case.

#### 3.2.1. Drug-Specific Anti-Angiogenesis Profiling

**Figure 3:**
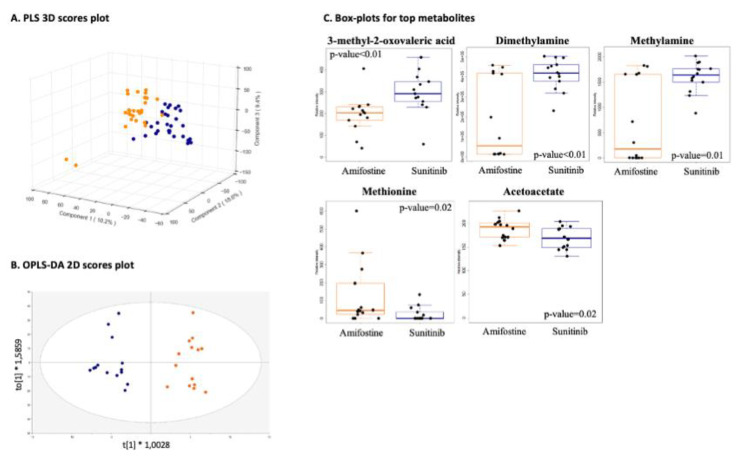
Drug-specific anti-angiogenesis profiling. (group comparison-c; amifostine_deferoxamine_VEGF-A, *n* = 28 vs. sunitinib_deferoxamine_VEGF-A, *n* = 28). (**A**) PLS 3D scores with R2 greater than 0.9 and Q2 equal to 0.8 using five components; (**B**) OPLS-DA 2D scores plot; (**C**) boxplots of top metabolites and their *p*-values. Group comparisons are color-coded; orange, angiogenesis stimulation and blue, angiogenesis inhibition.

Metabolomic profiles showed no major differences when amifostine was compared to sunitinib (group comparison-c) as expected, taking into account they both inhibited VEGF-A- or deferoxamine-induced angiogenesis, yet clusters per stimulant were evident, which was also as anticipated when considering different pro-angiogenic factors (Figure 3A,B). The amifostine metabolomes exhibited increased levels of methionine and acetoacetate, while sunitinib metabolomes showed higher levels of dimethylamine, methylamine, and 2-methyl-oxovaleric acid (Figure 3C).

Top molecular pathways (*p*-value < 0.05) include lysine and tyrosine metabolism, glycosphingolipid biosynthesis, and N-glycan degradation.

#### 3.2.2. Pathway-Specific Anti-Angiogenesis Profiling

**Figure 4:**
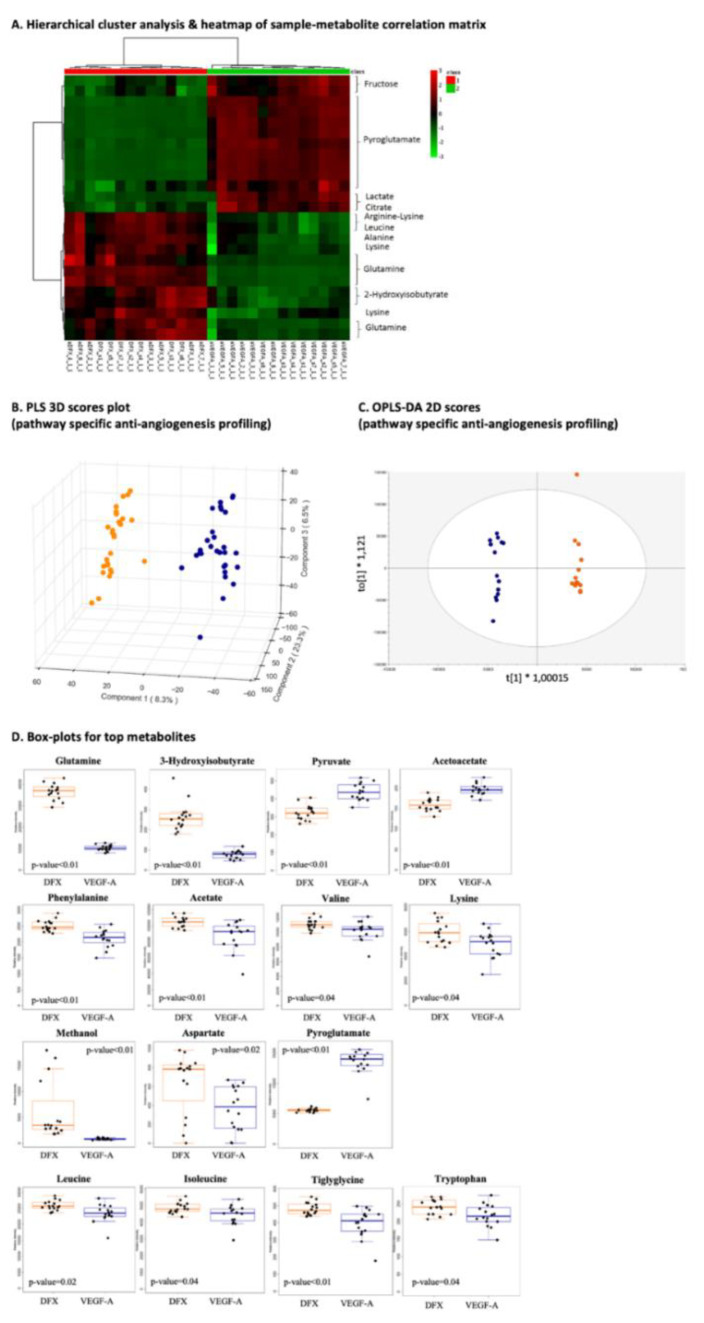
Pathway-specific anti-angiogenesis profiling. (**A**) Hierarchical cluster analysis and heatmap of sample–metabolite correlation matrix. Each square indicates the Pearson’s correlation coefficient of a pair of samples and metabolite(s) based on the analysis of variance values (group comparison-d; amifostine_sunitinib_deferoxamine, *n* = 28 vs. amifostine_sunitinib_ VEGF-A, *n* = 28). VEGF-A amifostine_1 cell extract is depicted and considered as an outlier as well as in the multivariate PCA models due to high glycerol concentration (the amount of glycerol distorts an NMR spectrum by affecting proton signal, resulting in a skewed NMR profile); (**B**) PLS 3D scores plot with R2 greater than 0.8 and Q2 equal to 0.8 using three components; (**C**) OPLS-DA 2D scores plot; (**D**) boxplots of top metabolites and their *p*-values (group comparisons are color-coded; orange, angiogenesis stimulation and blue, angiogenesis inhibition).

Great differences arose from VEGF-A vs. deferoxamine profiles (group comparison-d) allowing for pathway-specific anti-angiogenesis profiling (Figure 4A–C), considering that both VEGF-A and deferoxamine are pro-angiogenic factors; nevertheless, deferoxamine promotes revascularization via the activation of vascular endothelial cell function by an Akt-eNOS-dependent mechanism [39]. Pathway enrichment and analysis revealed aspartate and asparagine metabolism, urea cycle/amino group metabolism, and purine metabolism as the most prominent molecular pathways for the pro-angiogenic factors in question, and their action was clearly inhibited by amifostine or sunitinib. When compared to the VEGF-A metabolome, elevated levels of phenylalanine, glutamine, 3-hydroxyisobutyrate, aspartate, methanol, acetate, tiglylglycine, leucine, isoleucine, valine, lysine, and tryptophan were detected in the deferoxamine metabolome. The latter exhibited lower levels for pyroglutamate, pyruvate, aspartate, and acetoacetate when compared to the VEGF-A metabolome.

Top metabolites (*n* = 16) are summarized in Figure 4D.

#### 3.2.3. Anti-Angiogenesis Profiling Upon Deferoxamine Stimulation and/or Anti-Angiogenesis Profiling Upon VEGF-A

**Figure 5:**
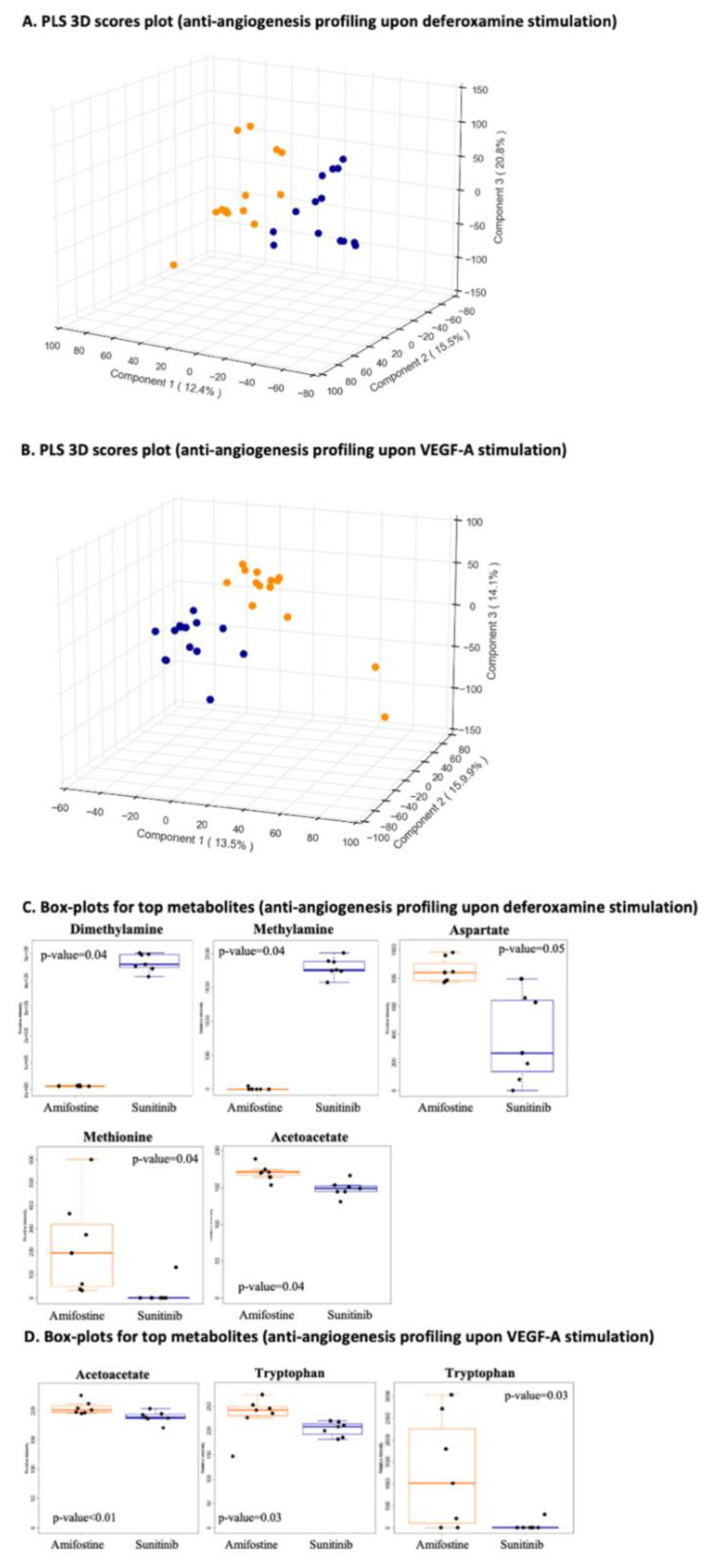
Stimulant-specific anti-angiogenesis profiling. (**A**) Anti-angiogenesis profiling upon deferoxamine stimulation (group comparison-a; amifostine_deferoxamine, *n* = 14 vs. sunitinib_deferoxamine, *n* = 14). PLS 3D scores plot with R2 greater than 0.9 and Q2 equal to 0.8 using five components; (**B**) anti-angiogenesis profiling upon VEGF-A stimulation (group comparison-b; amifostine_VEGF-A, *n* = 14 vs. sunitinib_VEGF-A, *n* = 14). PLS 3D scores plot with R2 greater than 0.9 and Q2 equal to 0.8 using five components; (**C**) boxplots of all the statistically significant metabolites and their *p*-values (FDR corrected) for anti-angiogenesis profiling upon deferoxamine stimulation (group comparison-a); (**D**) boxplots of all the statistically significant metabolites and their *p*-values (FDR corrected) for anti-angiogenesis profiling upon VEGF-A stimulation (group comparison-b). Group comparisons are color-coded; orange, angiogenesis stimulation and blue, angiogenesis inhibition.

For anti-angiogenesis profiling upon deferoxamine induction (group comparison-a) and/or anti-angiogenesis profiling after VEGF-A stimulation (group comparison-b), there are clearly distinct clusters between the two stimulants to angiogenesis (Figure 5A,B). Sunitinib and amifostine patterns were pretty similar and in accordance with their anti-angiogenic effects, yet distinct, implying that their mechanism-based inhibition profiles are not the same ones (Figure 5A,B). Taking into account that sunitinib is a kinase inhibitor, such findings are clearly expected.

Overall, the most prominent pathways for group comparison-a included the pentose phosphate pathway and purine metabolism, whereas lipoate metabolism and glycosphingolipid biosynthesis were the most prominent ones for group comparison-b. The top metabolites for group comparison-a are depicted in Figure 5C, while the ones for group comparison-b are shown in Figure 5D.

## 4. Discussion

Herein, we employed LC-MS-based and NMR-based untargeted 3D cell metabolomics to gain an insight into the anti-angiogenic effects of amifostine in adult angiogenesis as well as the first stages of the angiogenic cascade. Metabolomics can serve as a detailed snapshot of the overall status of cell physiology in a rapid and cost-efficient way, and, hence, data from two state-of-the-art technologies in metabolomics—liquid chromatography mass spectrometry and nuclear magnetic resonance spectroscopy—were acquired, processed, and analyzed [40,41]. We chose a 3D cellular angiogenesis assay, as 3D systems and models (cell culture, angiogenesis, etc.) better recapitulate cell physiology and, thus, drug action [21,42,43,44]. In particular, 3D culture models are considered as the optimum model for cell–cell and cell–matrix interactions [44].

To date, amifostine is widely used clinically as a radioprotectant and cytoprotectant to non-malignant tissues [45]. The active thiol metabolite (WR-1065) of amifostine shows higher levels in non-malignant tissues, and, overall, the selective radioprotection and cytoprotection of amifostine are well established [46]. The key question for us was to delineate the anti-angiogenic effects of amifostine in non-malignant tissues, as also previously described by us [13,14,15].

Angiogenesis is responsible for blood vessel growth in non-malignant (and malignant) tissues and is thought as a dynamic procedure with a series of processes that take place in a synchronous manner (to name a few, cell proliferation and migration, tube formation, specification into arteries, veins, and capillaries, etc.). The first event refers to cell sprouting upon their stimulation by pro-angiogenic factors (the most prominent stimulants to angiogenesis are VEGF-A and deferoxamine) [45,47]. This is the reason why we chose spheroids for our cellular angiogenesis assay upon VEGF-A or deferoxamine induction, while sunitinib served as a reference inhibitor. That said, herein, spheroids (hanging drops) were chosen as the most appropriate to address experimental challenges. To name a few, *n* = 3 cell lines, *n* = 2 cell culture conditions, *n* = 32 test conditions, *n* = 3 biological replicates (per test condition), and *n* = 3 technical replicates (per test condition).

Aberrant angiogenesis is a hallmark for cancer and inflammation, and, therefore, the discovery of anti-angiogenic chemical entities and drugs are of prime interest [48]. In the case of amifostine, which is already a drug on the market, we focus on drug repurposing and its greatly advantageous potential.

Our data shed light on the VEGF-A- and/or deferoxamine-induced anti-angiogenetic effects of amifostine. Although such drug action has been already reported both in vitro and in vivo [13,49], the exact mechanism remains unknown, while data on deferoxamine are scarce. Our findings highlight such mechanism-specific differences, while amifostine serves as an anti-angiogenic agent against VEGF-A- and deferoxamine-induced angiogenesis via the inhibition of cell sprouting. The metabolites identified in this study belong primarily in the categories of amino acids, sphingolipids, and nucleotides. Of note, this was an initial and sharp response, truly grasping the early events of angiogenesis. Next, targeted metabolomics may pave the way toward amifostine companion biomarkers.

## 5. Conclusions

Our study put emphasis on early angiogenesis events by coupling spheroids to LC-MS-based and NMR-based untargeted metabolomics. We report that amifostine exerts its anti-angiogenic effects against VEGF-A- and deferoxamine-induced angiogenesis via the inhibition of cell sprouting, also being an early sharp response. The 3D cell metabolomes delineate the molecular mechanisms involved during the inhibition of early events of angiogenesis, empowering drug repurposing and amifostine companion biomarker efforts.

## Data Availability

Data are available from D. Kardamakis and T. Katsila with the permission of Clinigen Group plc.

## Supplementary Materials

The following are available online at https://www.mdpi.com/article/10.3390/cancers13122877/s1, Table S1: Metabolites responsible for the differences between the angiogenesis stimulants, deferoxamine and VEGF-A (group comparison-d). Metabolites in bold characters are the statistically significant, Table S2: Metabolites responsible for the differences between the drug specific anti-angiogenesis profiles in question (group comparison-c). Metabolites in bold characters are the statistically significant.
