# Supplementary material for: Three-Dimensional Cell Metabolomics Deciphers the Anti-Angiogenic Properties of the Radioprotectant Amifostine
Source: Cancers (Basel). 2021 Jun 9;13(12):2877. doi: 10.3390/cancers13122877 (PMC8230228; doi:10.3390/cancers13122877)
Supplement: Supplementary file 1 [file cancers-13-02877-s001.zip › cancers-1228113-SI.pdf]

cancers MDPI

Supplementary Materials: Three-Dimensional Cell Metabolomics Deciphers the Anti-Angiogenic Properties of the Radioprotectant Amifostine

Table S1. Metabolites responsible for the differences between the angiogenesis stimulants, deferoxamine and VEGF-A (group comparison-d). Metabolites in bold characters are the statistically significant.

No	Metabolites	*p*. value	Change of relative concentration	Fold change	FDR adjusted
1	Pyroglutamate	1.41e-06	↑	-1.272	1.15e-05
2	Glutamine	1.41-06	↓	1.824	1.15e-05
3	3-Hydroxyisobutyrate	1.41-06	↓	1.777	1.15e-05
4	Methanol	1.41e-06	↓	2.41	1.15e-05
5	Pyruvate	6.11e-06	↑	-0.439	3.57e-05
6	Acetoacetate	5.11e-06	↑	-0.304	3.48e-05
7	Phenylalanine	7.58e-05	↓	0.264	0.0004
8	Acetate	0.0007	↓	0.298	0.0031
9	Tiglyglycine	0.0009	↓	0.315	0.004
10	Leucine	0.0047	↓	0.150	0.016
11	Aspartate	0.005	↓	NaN	0.015
12	Tryptophan	0.014	↓	0.170	0.037
13	Isoleucine	0.014	↓	0.147	0.037
14	Arabinose	0.014	↓	0.294	0.037
15	Valine	0.02	↓	0.139	0.044
16	Lysine	0.02	↓	0.279	0.044
17	Serine	0.0023	↓	NaN	0.09
18	3-Hydroxybutyrate	0.05	↑	-0.288	nd
19	3-methyl-2-oxovaleric acid	0.327	↑	-0.545	-
20	Lactate	0.678	↑	-0.002	-
21	Methylamine	0.272	↑	-inf	-
22	Methionine	0.258	↓	NaN	-
23	Dimethylamine	0.365	↑	-2.177	-
24	Choline	0.113	↑	-0.055	-
25	Lysine	0.0194	↓	0.279	-
26	Fructose	0.0382	↓	0.169	-
27	Threonine	1	↓	-Inf	-
28	4-hydroxyproline	0.651	↑	-0.122	-
29	Glucose	0.0648	↓	0.101	-
30	Allantoin	0.0704	↓	0.088	-
31	Fumarate	0.571	↓	0.066	-
32	Tyrosine	0.227	↓	0.113	-
33	Histidine	0.327	↓	0.105	-
34	π- Methylhistidine	0.734	↓	0.009	-
35	Formate	0.597	↓	0.046	-
36	Niacinamide	0.407	↑	-0.039	-

Table S2. Metabolites responsible for the differences between the drug specific anti-angiogenesis profiles in question (group comparison-c). Metabolites in bold characters are the statistically significant.

No	Metabolites	*p.* value	Change of relative concentration	Fold change	FDR adjusted
1	3-methyl-2-oxovaleric acid	0.002	↑	-0.645	-
2	Dimethylamine	0.008	↑	-2.573	-
3	Methylamine	0.013	↑	-inf	-
4	Methionine	0.023	↓	nan	-
5	Acetoacetate	0.027	↓	0.157	-
6	Leucine	0.383	↓	0.008	
7	Valine	0.78	↑	-0.017	
8	Isoleucine	0.96	↑	-0.015	
9	3-Hydroxybutyrate	0.183	↑	-0.180	
10	2-Hydroxyisobutyrate	0.71	↓	0.071	
11	Alanine	0.78	↑	-0.019	
12	Tiglyglycine	0.89	↑	-0.045	
13	Acetate	0.81	↑	-0.075	
14	Glutamine	1	↑	-0.002	
15	Pyruvate	0.78	↓	0.025	
16	Aspartate	0.2	↓	NaN	
17	Lysine	0.29	↓	0.034	
18	Choline	0.78	↑	-0.047	
19	Methanol	0.81	↓	0.29	
20	Serine	0.58	↓	NaN	
21	Fructose	0.93	↑	-0.034	
22	Pyroglutamate	0.68	↓	0.077	
23	Threonine	0.61	↓	Inf	
24	4-Hydroxyproline	0.098	↓	0.053	
25	Arabinose	0.29	↓	0.077	
26	Glucose	0.85	↑	-0.021	
27	Allantoin	0.25	↑	-0.048	
28	Fumarate	0.65	↑	-0.085	
29	Tyrosine	0.10	↓	0.043	
30	Phenylalanine	0.18	↓	0.059	
31	Tryptophan	0.11	↓	0.073	
32	Histidine	0.27	↓	0.027	
33	π-Methylhistidine	0.27	↑	-0.056	
34	Formate	1	↓	0.038	
35	Niacinamide	0.55	↑	-0.069	
36	Lactate	0.38	↓	0.035	
